# Impaired Expression of Insulin-Like Growth Factor-1 System in Skeletal Muscle of Amyotrophic Lateral Sclerosis Patients

**DOI:** 10.1002/mus.22288

**Published:** 2012-02

**Authors:** Christian Lunetta, Massimo Serafini, Alessandro Prelle, Paolo Magni, Elena Dozio, Massimiliano Ruscica, Jenny Sassone, Clarissa Colciago, Maurizio Moggio, Massimo Corbo, Vincenzo Silani

**Affiliations:** 1 Department of Neurology and Laboratory of Neuroscience, “Dino Ferrari” Center, IRCCS Istituto Auxologico Italiano, Università degli Studi di MilanoMilano, Italy; 2 Neuromuscular Omnicentre, Fondazione Serena Onlus, Niguarda Ca' Granda HospitalPiazza Ospedale Maggiore 3, Milano, Italy; 3 Dino Ferrari Center, University of Milan, IRCCS Foundation Ca' Granda Ospedale Maggiore PoliclinicoMilano, Italy; 4 U.O. di Neurologia, Dipartimento di Neuroscienze, Azienda Ospedaliera Fatebenefratelli e OftalmicoMilano, Italy; 5 Department of Endocrinology, Pathophysiology and Applied Biology, Università degli Studi di MilanoMilano, Italy; 6 Department of Human Morphology and Biomedical Sciences “Citt' Studi,” Università degli Studi di MilanoMilano, Italy

**Keywords:** amyotrophic lateral sclerosis, IGF-1, IGF-BPs, IGF-1 receptor, skeletal muscle

## Abstract

**Introduction:**

Adult muscle fibers are a source of growth factors, including insulin-like growth factor-1 (IGF-1). These factors influence neuronal survival, axonal growth, and maintenance of synaptic connections.

**Methods:**

We investigated the components of the IGF system in skeletal muscle samples obtained from 17 sporadic amyotrophic lateral sclerosis patients (sALS) and 29 control subjects (17 with normal muscle and 12 with denervated muscle unrelated to ALS).

**Results:**

The muscle expression of IGF-1 and IGF-binding proteins 3, 4, and 5 (IGF-BP3, -4, and -5, respectively), assessed by immunohistochemistry, was differently decreased in sALS compared with both control groups; conversely, IGF-1 receptor β subunit (IGF-1Rβ) was significantly increased. Western blot analysis confirmed the severe reduction of IGF-1, IGF-BP3, and -BP5 with the increment of IGF-1Rβ in sALS.

**Conclusion:**

In this study we describe the abnormal expression of the IGF-1 system in skeletal muscle of sALS patients that could participate in motor neuron degeneration and should be taken into account when developing treatments with IGF-1. Muscle Nerve, 2012

Amyotrophic lateral sclerosis (ALS) is a fatal neuromuscular disorder characterized by motor neuron (MN) degeneration that leads to progressive skeletal muscle atrophy and paralysis. Most ALS cases are sporadic, but a small percentage (5–10%) are familial.

Many hypotheses have been formulated to explain the pathogenesis of sporadic ALS (sALS), including autoimmune reactions to calcium channels on MNs, glutamate-induced excitotoxic injury, exposure to toxins or latent infections, disorganization of intermediate filaments, and loss of neurotrophic support to MNs.^1^ The latter hypothesis is of particular interest, because adult muscle fibers produce molecules that influence MN survival, axonal growth, and maintenance of synaptic connections. Among these trophic factors, insulin-like growth factor-1 (IGF-1) has a key role; it is involved in muscle and nerve tissue anabolism and thus induces muscle hypertrophy and promotes neuronal survival.^2^–^6^ The neurotrophic effect of IGF-1 was the starting point for three clinical trials based on subcutaneous injections of human recombinant IGF-1 in ALS patients. Disappointingly, these studies did not show a significant positive effect of IGF-1 therapy on disease progression or survival in ALS patients.^7^–^9^ However, the therapeutic role of IGF-1 in ALS is still debated. Indeed, Kaspar and colleagues demonstrated that treatment with adeno-associated virus/IGF-1 retrogradely transported from muscle to MNs of the spinal cord led to therapeutic benefits in the G93A transgenic mouse model.^10^ This effect was further increased with associated physical exercise.^11^

More recently, Dobrowolny et al. reported that muscle-restricted expression of IGF-1 isoforms maintained muscle integrity, stabilized neuromuscular junctions, reduced inflammation in the spinal cord, enhanced motor neuronal survival, and delayed the onset and slowed disease progression in sodium dismutase 1 (SOD1) G93A mice.^12^, ^13^ Both these studies reappraised the potential role of the skeletal muscle and IGF-1 signaling as a target for treatment in ALS patients. Moreover, a recent study also showed that overexpression of IGF-1 in muscle attenuates disease in a mouse model of spinal and bulbar muscular atrophy.^14^

The IGF-1 signaling system is complex and regulated by many factors.^15^, ^16^ The components of the IGF-1 system include a growth factor, IGF-1, which is a single-chain polypeptide with a molecular weight of approximately 7.5 kDa. The IGF-1 receptor (IGF-1R) is a membrane glycoprotein of 300–350 kDa. It consists of two α subunits (135 kDa each) containing the ligand-binding site, two β subunits (90 kDa each) containing the hydrophobic transmembrane domain, a short extracellular region, and a tyrosine kinase domain in its cytoplasmic portion.^17^ Besides IGF-1R, six IGF-binding proteins (IGF-BPs) have been identified. This is a family of secreted proteins that specifically bind IGF-1 with affinities that are equal to or greater than those of the IGF-1R. In terms of IGF-1 function, it is now well established that some of the IGF-BPs, such as -BP2, -BP4, and -BP6, inhibit IGF-1. BP5 potentiates IGF-1 actions, whereas -BP1 and -BP3 can inhibit or potentiate IGF-1.^18^–^22^ In addition, numerous data also demonstrate evidence for IGF-1–independent actions of IGF-BPs, which are unexpected.^23^, ^24^

The lack of information available on the expression levels of the IGF-1 system in skeletal muscle of ALS patients led us to investigate the expression of IGF-1; IGF-BP3, -BP4, -BP5; and IGF-1R β subunit (IGF-1Rβ) in skeletal muscle specimens and primary muscle cell cultures obtained from sALS patients.

## METHODS

### Clinical Data for Patients and Control Subjects

We enrolled 17 sALS patients recruited from the Department of Neurology of the IRCCS Istituto Auxologico Italiano (Milan, Italy). The diagnosis of sALS was based on a detailed history and physical examination, supported by electrophysiological evaluation.^25^ Other disease causes were excluded by appropriate blood tests and neuroimaging.

The sALS group included 15 men and 2 women (mean age 55.2 ± 11.5 years), and the patients were classified according to the revised El Escorial criteria^26^ in the following groups: definite ALS (*n* = 6 patients); probable ALS (*n* = 8); and possible ALS (*n* = 3). Thirteen sALS patients presented with limb onset and 4 with bulbar onset. The mean Amyotrophic Lateral Sclerosis Functional Rating Scale—revised (ALS-FRS-R) score was 32.8 (±9.1), and disease duration ranged from 3 to 96 months (median = 11 months). All patients had been taking riluzole for ≥6 months.

The control group included 17 subjects with normal muscle at histological analysis (healthy controls, HCs), divided into 10 men and 7 women (mean age 54.6 ± 14.6 years), and 12 subjects with non–ALS-related denervation (pathologic controls, PCs), divided into 7 men and 5 women (mean age 54.1 ± 12.1 years). The PC patients included the following: chronic inflammatory demyelinating polyneuropathy (*n* = 6); spondylogenic radiculopathy (*n* = 2); and multifocal motor neuropathy (*n* = 4).

The nutritional status, evaluated by body mass index (BMI = weight/height^2^) and serum nutritional parameters (such as total protein, pre-albumin, and transferrin), was similar in sALS patients and controls (*P* > 0.05). Subjects with diabetes mellitus were excluded from the study. Open biopsy was performed in the biceps muscle in all subjects.

This investigation was approved by the local ethics committee, and patients signed an informed consent document consistent with institutional guidelines.

### Histochemical and Immunofluorescence Analyses

Specimens were frozen in isopentane–liquid nitrogen and stored in liquid nitrogen until use. Histological, histochemical, and immunoflourescence (IF) analyses were carried out on 8-μm-thick frozen cross-sections. Monoclonal antibodies for IGF-1 and IGF-BP3, -BP4, and -BP5 were supplied by R&D Systems (Minneapolis, Minnesota), and antibodies for IGF-1Rβ were from Upstate Biotechnology (Lake Placid, New York). For IGF-1 and IGF-BP3, -BP4, and -BP5 analyses, muscle sections were fixed in acetone for 1 min at 4°C and blocked with normal goat serum in phosphate-buffered saline (PBS). For IGF-1Rβ analysis, tissues were fixed with 4% paraformaldehyde for 15 min at room temperature and permeabilized with 0.01% Triton/PBS for 5 min at room temperature. Sections were incubated at 4°C overnight with the primary antibodies and then incubated with secondary antibodies. Secondary biotinylated anti-mouse antibodies were applied for 30 min (1:100; Amersham Life Sciences), followed by streptavidin–fluorescein for 30 min (1:100; Amersham). In the case of IGF-1Rβ, secondary Cy2-conjugated goat anti-mouse IgGs were applied for 1 h (1:200; Jackson ImmunoResearch, Inc., West Grove, Pennsylvania). All incubations were carried out at room temperature in a wet chamber. As negative controls, primary antibodies were omitted. Sections were examined under a Zeiss fluorescence microscope. The studies were analyzed in a blinded fashion by two different investigators (A.P. and M.M.).

To compare the degree of denervation between sALS and PC skeletal muscles, we used a denervation index according to Brooke et al.^27^ Briefly, the index was calculated from the histograms of the muscle fibers and provided the number of abnormally small fibers in the biopsy (denervated fibers). The parameter was obtained by multiplying the number of fibers in the histogram with a diameter between 30 and 40 μm by 1, the number of fibers with a diameter between 20 and 30 μm by 2, the number of those between 10 and 20 μm by 3, and the number in the group <10 μm by four. These products were then added together and divided by the total number of fibers in the histogram to show the result on a proportional basis. Finally, the resulting number was multiplied by 1000.

### Western Immunoblotting Analysis

Samples of frozen muscle were homogenized in ice-cold lysis buffer [0.05 M Tris-HCl, 0.15 M NaCl, 0.8% Triton X-100, 0.08% sodium dodecylsulfate (SDS), 10 mM ethylene-diamine tetraacetic acid, 100 μM sodium vanadate, 0.8% sodium deoxycholate, 50 mM sodium floride, 5 mM iodoacetic acid] containing 1% protease inhibitor cocktail (Sigma-Aldrich, Milan, Italy). The homogenate was kept on ice for 30 min, centrifuged at 500 rpm for 10 min at 4°C, and the resulting supernatant centrifuged at 13,200 rpm for 15 min at 4°C. Protein concentration was determined with the bicinchoninic acid (BCA) assay (Pierce, Rockford, Illinois). Blood contamination was excluded by showing the absence of albumin—measured with an albumin immune assay (BN2; Behring, Marburg, Germany).

For investigation of IGF-1, IGF-BP, and IGF-1Rβ expression, equal amounts of protein samples (50 μg) were resuspended in Laemmli sample buffer and separated in an 8–16% Tris–HEPES–SDS-polyacrylamide gel system (Pierce). The separated proteins were then transferred from the gel to a nitrocellulose membrane overnight at 4°C. The membrane was blocked with 5% dry milk in Tris-buffered saline/0.1% Tween 20 for 1 h at room temperature, and the blot was then incubated overnight at 4°C with a diluted primary antibody solution of anti–IGF-1 (1:500), anti–IGF-BP3 (1:500), anti–IGF-BP4 (1:500), anti–IGF-BP5 (1:500) (all from R&D Systems), or anti–IGF-1Rβ (1:1000; Cell Signaling Technology, Danvers, Massachusetts). The subsequent incubation with a secondary antibody conjugated with peroxidase was performed at room temperature for 2 h. Immunoreactivity was detected by working solution (SuperSignal West Pico Substrate; Pierce) and exposure of the membrane to photographic film at room temperature for the required time.

### Primary Muscle Cell Cultures

Primary adult skeletal muscle cell cultures were obtained as previously described.^28^ IGF-1Rβ expression was evaluated in cell cultures from 2 sALS patients and 2 healthy, age-matched controls. Membrane protein fractions were obtained by solubilization in ice-cold lysis buffer for 1 h at 4°C and subsequent centrifugation for 1 h at 4°C at 100,000 × *g*. Protein quantification and WB analysis were then conducted as described earlier.

### Statistical Analysis

Statistical analysis for WB analysis was performed using Prism statistical analysis software (GraphPad Software, San Diego, California). Data are given as mean ± SD. Differences between groups were evaluated by analysis of variance (ANOVA), followed by *post hoc* Tukey and Dunnett tests and considered significant at *P* < 0.05.

## RESULTS

### Histochemical Analysis of Skeletal Muscle

In all ALS patients, histopathological examination showed a typical chronic neurogenic pattern, with groups of atrophic and angulated fibers associated with diffuse fiber-type grouping. The atrophic fibers were present in large or small groups and were of both histochemical fiber types. The small, angulated fibers were intensely stained with oxidative enzymes. Target fibers and central nuclei were also present. Moreover, we selected PC patients looking for muscle biopsies with a similar degree of pathological changes, particularly chronic denervation. The degree of denervation was similar between ALS and PC patients according to histometrical analysis (Table 1).

**Table 1:** Histometrical analysis of denervation index in sALS and PC skeletal muscle

	sALS (*n* = 17)	PC (*n* = 12)	*P*
Type 1 fibers (mean ± SD)	1025 ± 645	1234 ± 913	0.48
Type 2A and type 2B fibers (mean ± SD)	1139 ± 782	1191 ± 782	0.87

PC, pathologic control.

### IF Analysis of IGF-1 System in Skeletal Muscle

**FIGURE 1:**
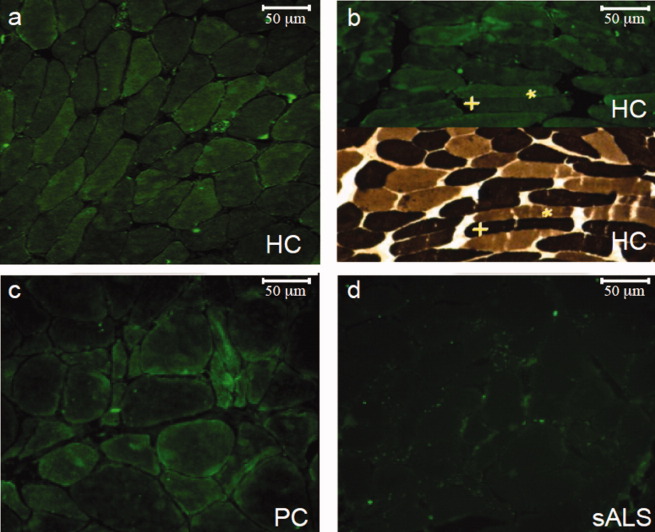
Histochemical and IF studies of IGF-1 in skeletal muscle. (**a**) Immunoreactivity for IGF-1 in HC muscles had a cytoplasmic distribution characterized by a “checkerboard” pattern. (**b**) The 9.4 ATPase reaction in HC muscles showed that type II fibers (+) had weaker immunostaining compared with type I fibers (*). (**c**) Although the muscle fibers were differently immunoreactive for IGF-1, the checkerboard pattern was still evident in PC muscles. (**d**) In sALS patients, IGF-1 immunostaining was definitely weak, and the checkerboard pattern was totally lost (muscle cryosections; bar = 50 μm). IF, immunofluorescence; HC, healthy control; PC, pathological control. [Color figure can be viewed in the online issue, which is available at wileyonlinelibrary.com.]

**FIGURE 2:**
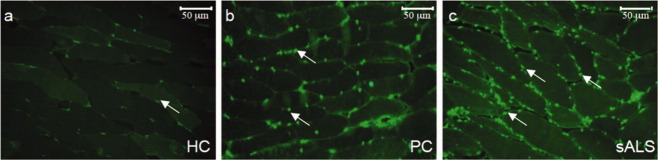
IF studies of IGF-1-Rβ in skeletal muscle. (**a**) In HC muscles, receptor β had a selective distribution in a spotty pattern on the sarcolemma. (**b, c**) In PCs and sALS muscles, IGF-1-Rβ immunostaining showed increased reactivity compared with HCs; however, this was more intense in the muscles of sALS patients (muscle cryosections; bar = 50 μm). IF, immunofluorescence; HC, healthy control; PC, pathological control. [Color figure can be viewed in the online issue, which is available at wileyonlinelibrary.com.]

**FIGURE 3:**
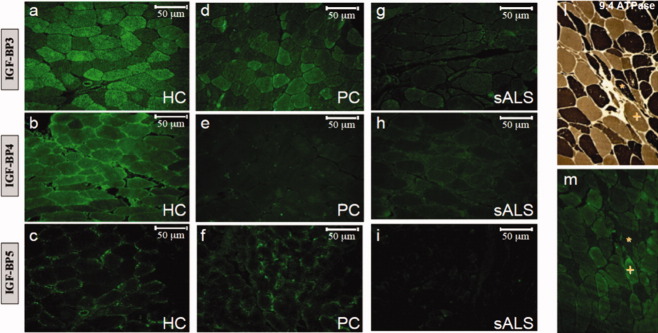
Histochemical and IF studies of IGF-BPs in skeletal muscle. (**a–c**) In HCs, all three IGF-BPs displayed a cytoplasmic distribution. In particular, immunoreactivity for both IGF-BP3 (**a**) and IGF-BP5 (**c**) showed a “checkerboard” pattern, in which type II muscle fibers (**l,m**) (*) showed weak immunostaining compared with type I fibers (**l, m**) (+). The signal was uniform inside the muscle fibers for IGF-BP3 (**a**), higher next to the muscle fiber surface for IGF-BP4 (**b**), and scattered for IGF-BP5 (**c**). In PC tissues, the immunoreactivity of the three IGF-BPs (**d–f**) was slightly weaker as compared with HC tissues, whereas the distribution pattern did not differ between the two control groups. In skeletal muscle of sALS patients, expression of the investigated BPs (**g–i**) was significantly decreased compared with the control groups (muscle cryosections; bar = 50 μm). IF, immunofluorescence; HC, healthy control; PC, pathologic control. [Color figure can be viewed in the online issue, which is available at wileyonlinelibrary.com.]

Normal skeletal muscle fibers (HC) showed specific signal for IGF-1, IGF-1Rβ, and all three IGF-binding proteins (BP3, BP4, and BP5). Immunoreactivity for IGF-1 in HC muscles showed a cytoplasmic distribution characterized by a “checkerboard” pattern (Fig. 1a): type II muscle fibers had weaker immunostaining when compared with type I muscle fibers (Fig. 1b). In addition, IGF-1Rβ reactivity showed a spotty distribution on the sarcolemma of HC muscle fibers (Fig. 2a). All three IGF-BPs displayed a cytoplasmic location in the muscle fibers of HC, but some differences were evident among them. IGF-BP3 and -BP5 were differently expressed in these muscles, showing a fiber type–related distribution (Fig. 3a and c). The immunobinding was uniformly evident inside the muscle fibers for IGF-BP3, whereas it was speckled and more prominent in the outer region of cytoplasm for IGF-BP5. IGF-BP4 had a cytoplasmic expression, more concentrated next to the sarcolemma, without a specific fiber-type distribution (Fig. 3b).

In PC tissues, the IGF-1 and its BPs were expressed (Fig. 1c and 3d–f), but their immunoreactivity was slightly weaker when compared with HC muscles. The distribution pattern did not differ between the two control groups. On the contrary, IGF-1Rβ immunobinding was more intensely expressed than in HC tissues (Fig. 2b).

In skeletal muscle of sALS patients the immunostaining of IGF-1 and IGF-BP3, -BP4, and -BP5 (Figs. 1d and 3g–i) was decreased when compared with the control groups. In particular, the reduction of expression was more evident for IGF-1 and IGF-BP3 and -BP5. Interestingly, the “checkerboard” pattern observed for IGF-1 and IGF-BP3 and -BP5 was totally lost. Notably, in sALS patients, immunostaining showed a remarkable increment of IGF-1Rβ spot reactivity at the level of the sarcolemma as compared with both healthy and pathological controls (Fig. 2c).

### WB Analysis of IGF-1 System in Skeletal Muscle

**FIGURE 4:**
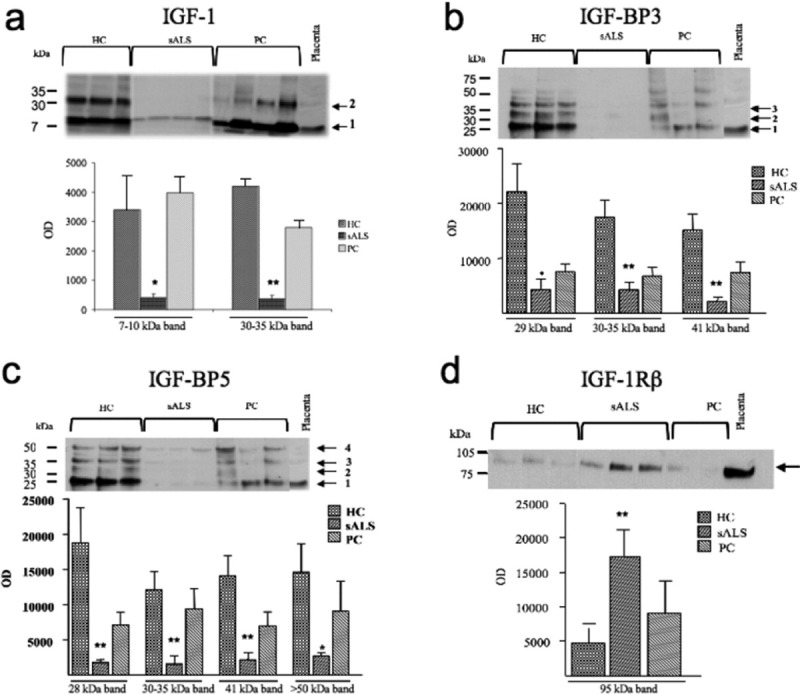
Representative WB experiment and densitometric analysis (mean ± SD) of IGF-1 (**a**), IGF-BP3 (**b**), IGF-BP5 (**c**), and IGF-1Rβ (**d**) expression for HC, sALS, and PC skeletal muscle. ^*^P < 0.05 vs. HC; ^**^P < 0.01 vs. HC and PC. WB, Western blot; HC, healthy control; PC, pathologic control.

**FIGURE 5:**
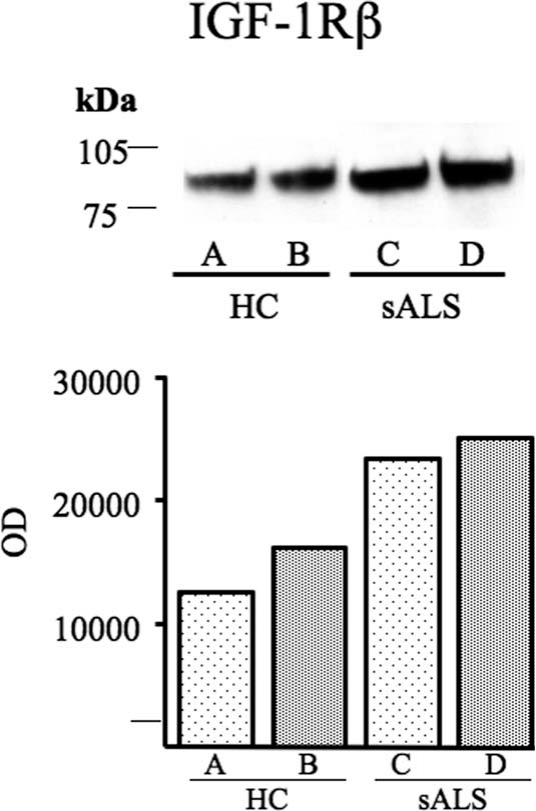
WB analysis of IGF-1Rβ expression in primary adult skeletal muscle cell cultures from 2 sALS patients and 2 age-matched HCs (A control of C and B control of D). Densitometric analysis is also shown. WB, Western blot; HC, healthy control.

WB analysis of skeletal muscle from HCs showed two main IGF-1 immunoreactive bands, one of 25–30 kDa corresponding to bound IGF-1, and another <10 kDa, possibly free IGF-1.^29^ The intensity of both signals was markedly lower in sALS muscle homogenates compared with those from HC and PC (Fig. 4a and Table 2). WB analysis of IGF-BP3 expression yielded a main band of 29 kDa and additional 30–35- and 41-kDa bands, corresponding to putative glycosylated forms (Fig. 4b and Table 2). WB analysis of IGF-BP5 showed a main band of 28 kDa and other bands of 30–35, 41, and >50 kDa (Fig. 4c and Table 2). Immunoreactivity for both IGF-BP3 and -BP5 was dramatically lower in all sALS homogenates compared with controls. No significant differences were detected for IGF-BP4 in the muscle samples from all three groups (data not shown). The IGF-1Rβ signal was observed as a ∽95-kDa band that was more intense in sALS muscle tissues compared with HC and PC tissues (Fig. 4d and Table 2). To confirm the increased IGF-1Rβ expression observed in sALS, we evaluated the receptor immunoreactivity in primary adult skeletal muscle cell cultures from 2 sALS patients and 2 healthy, age-matched controls. The analysis of protein-enriched membrane samples showed that sALS cells expressed more IGF-1Rβ than control cells (Fig. 5: C vs. A and D vs. B), corroborating evidence of an increment of this growth factor receptor in sALS skeletal muscles. Analysis of receptor immunoreactivity in primary adult skeletal muscle cell cultures of PC was not performed.

**Table 2:** Densitometric WB analysis of IGF-1, IGF-BP3, IGF-BP5, and IGF-1Rβ in HC, sALS, and PC skeletal muscle

	HC	sALS	PC
IGF-1			
7–10-kDa band	3933 ± 3057	399 ± 115*	3973 ± 1125
30-kDa band	4210 ± 542	367 ± 101†	2792 ± 564
IGF-BP3			
29-kDa band	22,061 ± 5030	4291 ± 1932*	7490 ± 1471
30–35-kDa band	17,445 ± 3080	4177 ± 1775†	6712 ± 1687
41-kDa band	15,067 ± 2970	2171 ± 796†	7413 ± 1858
IGF-BP5			
28-kDa band	18,697 ± 4998	1742 ± 460†	7146 ± 1788
30–35-kDa band	12,158 ± 2548	1613 ± 410†	9404 ± 2852
41-kDa band	14,145 ± 2759	2207 ± 637†	6968 ± 2011
>50-kDa band	14,593 ± 4078	2668 ± 539*	9082 ± 4232
IGF-1Rβ			
95-kDa band	4778 ± 2078	17,392 ± 3822†	9081 ± 4726

Data are presented as mean ± SD. WB, Western blot; HC, healthy control; PC, pathologic control.

* *P* < 0.05 vs. HC.

† *P* < *0.01 vs. HC and PC.*

## DISCUSSION

IGF-1 is a trophic factor for different tissues, including nervous system and skeletal muscle. In particular, it is a survival factor for motoneurons, both *in vitro* and *in vivo*.^2^, ^30^–^32^ It is also a potent myogenic factor that promotes myoblast proliferation, myogenic differentiation, and myotube hypertrophy.^2^–^6^ In this study, in which IF and WB analyses were utilized, some IGF-1 system components, represented by IGF-1 and three binding proteins (BP3, BP4, and BP5), were less expressed in skeletal muscle in sALS patients than in normal and pathological controls. On the contrary, the muscle expression of IGF-1Rβ was increased in sALS patients compared with controls. These data might reflect a worrisome inhibition of the action of the IGF-1 system in skeletal muscles of sALS patients that could influence the course of the disease.

Wilczak et al. investigated the components of the IGF-1 system in spinal cord sections of patients with ALS.^29^ They reported that free IGF-1 concentrations were lower in ventral horn homogenates from ALS patients than in those from controls; in addition, IGF-1R immunoreactivity was enhanced in ALS spinal MNs. Indeed, our study disclosed a similar dysfunction of the IGF-1 system in another compartment of sALS patients that could play an important role in both MN degeneration and muscle atrophy. On the other hand, Millino et al. found that patients with spinal muscular atrophy type I, a neurodegenerative disorder associated with mutations of the survival MN gene and characterized by muscle weakness and atrophy caused by degeneration of spinal MNs, showed reduced expression of the genes involved in the IGF/PI3K/Akt pathway with an overexpression of the IGF-1R gene.^33^

Several hypotheses on the cause of this reduced activation of the IGF-1 system should be considered. First, nutrition is one of the main regulators of circulating IGF-1, so low nutrient intake downregulates IGF-1 gene transcription^34^ and reduces serum IGF-1 concentrations. The exquisite sensitivity of circulating IGF-1 to nutrients, the nycthemeral stability of its concentrations, and its relatively short half-life constitute the basis for its use as a marker of both nutritional status and adequacy of nutritional rehabilitation. Mazzini et al. reported that, at the onset of medical attendance, 53% of their ALS patients had a BMI <20 kg/m^2^, and 55% had a weight loss of >15% of their usual weight. These data indicate a high occurrence of malnutrition in ALS patients that leads to an impairment of muscle function eventually mediated by reduced IGF-1 availability.^35^ This was not the case for the patients we studied, because they did not show any alteration of parameters of nutritional status.

A second hypothesis may be related to the role of cytokines in ALS. Accumulating evidence from studies in both cell cultures and ALS animal models suggests that the pro-inflammatory cytokine, tumor necrosis factor-α (TNF-α), may participate in the death of MNs, even from the early pathogenic events of the neurodegenerative process.^36^, ^37^ Both serum TNF-α and its soluble receptors have been reported to be significantly higher in ALS patients compared with healthy controls.^38^ Moreover, TNF-α impairs skeletal muscle trophism. In this context, activation of TNF-α signaling via the c-Jun N-terminal kinase can decrease IGF-1 RNA expression and inhibit IGF-1 signaling by phosphorylation and conformational changes in insulin receptor substrate-1 downstream of the IGF-1R.^39^

A third possible reason that may influence the IGF-1 system is related to growth hormone (GH). This important anabolic hormone has direct and indirect actions on protein synthesis of different tissues, including skeletal muscle.^40^, ^41^ Importantly, the indirect effects of GH are mediated mainly through the production of IGF-1.^42^, ^43^ Morselli et al. found that the majority (73%) of ALS patients have a GH deficiency, and thus this condition could induce a reduction of tissue IGF-1 synthesis.^44^

Finally, we considered that the initial response of the IGF-1 receptor to its ligand is related to an autophosphorylation on specific tyrosine residues. The intrinsic tyrosine kinase activity of IGF-1R phosphorylates multiple substrates that determine the activation of several downstream molecules, such as the serine–threonine kinase Akt. Thus, Leger et al. observed reduced active Akt in skeletal muscle of ALS patients and G93A SOD1 transgenic mice, which may be the “tip of the iceberg.”^45^ In other words, the reduction of Akt coincides with the decreased expression of IGF-1 and the increased expression of IGF-1R that we observed.

According to morphological criteria and results of histometrical analysis the degrees of denervation were similar between sALS and PC patients. These data support the idea that the augmented expression of IGF-1R in ALS skeletal muscles is not an effect of denervation but could be a specific phenomenon in sALS patients.

In this scenario it is also worth considering the relevant role of BPs in the balance of effects caused by the IGF-1 system components. IGF-BP3 is the most abundant circulating BP and exerts a complex array of functions at the cellular level. Primarily, IGF-BP3 inhibits IGF-1–mediated effects via high-affinity sequestration of the ligand, presumably leading to prevention of IGF-1R autophosphorylation and signaling.^18^ It has also been reported, based on competitive ligand-binding studies, that IGF-BP3 can interact with IGF-1R, causing inhibition of IGF-1 binding to its receptor.^46^

However, the recombinant human (rh)IGF-1/IGF-BP3 complex, which improves the safety and efficacy profile of rhIGF-1 alone,^47^ has an anabolic potential demonstrated in catabolic conditions such as burn injuries.^48^ In an *ad hoc* animal study, the IGF-1/IGF-BP3 complex, but not IGF-1 alone, was able to support muscle protein synthesis in rats during semi-starvation.^49^ IGF-BP5 is considered a stimulatory BP because it seems to counteract the inhibitory actions of other BPs, such as IGF-BP4, in systems such as bone^50^ and cultured vascular smooth muscle cells.^51^ It is noteworthy that, in this study, we observed a reduced expression of IGF-1 and IGF-BP3 and -BP5, which could suggest a negative interference of muscle anabolism in ALS patients, even when considering the unchanged IGF-BP4 content in their muscle.

Previously, we demonstrated a high level of the active fraction of IGF-1 (free IGF-1) in the cerebrospinal fluid (CSF) of sALS patients without any change in serum and CSF levels of total IGF-1.^52^ In the present study we found a distinctive expression of IGF-1 and IGF-BP3 and -BP5 according to muscle fiber types, with a higher expression level of slow-twitch (ST) or type I fibers compared with fast-twitch (FT) or type II fibers (checkboard pattern). In light of this distribution, which was previously shown only in rat skeletal muscle fibers for the two BPs,^53^ the different resistance to denervation by the two types of muscle fibers should be considered. Pun et al. showed that in two mouse models of motoneuron disease (G93A SOD1 and G85R SOD1), axons of fast-fatigable motoneurons are affected synchronously, long before symptoms appear.^54^ Thus, fast-fatigue–resistant motoneuron axons are affected at symptom onset, whereas axons of slow motoneurons are resistant.

In conclusion, ALS is emerging as a “multisystemic” disease in which structural, physiological, and metabolic alterations in different tissues or cell types (motoneurons, glia, and muscle fibers) may act synergistically to induce and/or exacerbate the disease.^55^, ^56^ The cell interaction represents a functional cross-talk between neuronal and non-neuronal cells, whose derangement could make some therapeutic strategies ineffective. Therefore, although dysfunctions in the IGF-1 system emphasize the potential therapeutic role of IGF-1 in sALS, we should be aware that some disease-related tissue abnormalities can interfere with the potential therapeutic properties of this neurotrophic factor.

## Abbreviations

ALS: amyotrophic lateral sclerosis
ANOVA: analysis of variance
BCA: bicinochininic acid
BMI: body mass index
CSF: cerebrospinal fluid
FT: fast twitch
GH: growth hormone
HC: healthy controls
IGF-1: insulin-like growth factor-1
IGF-BP: IGF-binding protein
IF: immunofluorescence
MN: motor neuron
PBS: phosphate-buffered saline
PC: pathological control
rh: recombinant human
sALS: sporadic amyotrophic lateral sclerosis
SOD1: sodium dismutase 1
SDS: sodium dodecylsulfate
ST: slow twitch
TNF-α: tumor necrosis factor-α
WB: Western blot
